# Development and psychometric evaluation of a theory-based questionnaire measuring women’s return-to-work beliefs after long-term sick leave for common mental disorders

**DOI:** 10.3233/WOR-220301

**Published:** 2023-09-11

**Authors:** Åsa Hedlund, Eva Boman, Marja-Leena Kristofferzon, Annika Nilsson

**Affiliations:** a Department of Caring Sciences, University of Gävle, Gävle, Sweden; b Department of Occupational Health Science and Psychology, University of Gävle, Gävle, Sweden

**Keywords:** Mental health recovery, psychological theory, psychometrics, rehabilitation

## Abstract

**BACKGROUND:**

Common mental disorders (CMDs) are currently a major cause of long-term sick leave, with women being most affected.

**OBJECTIVE:**

Using the Theory of Planned Behaviour (TPB), we aimed to describe the development and psychometric evaluation of a new questionnaire to measure women’s beliefs about return to work (RTW) after long-term sick leave for CMDs.

**METHODS:**

Data were collected in central Sweden from women on long-term sick leave (2– 24 months) for CMDs. The questionnaire was developed by conducting an elicitation study with 20 women and included both direct and indirect measures. Subsequently, 282 women participated in a psychometric evaluation and 35 of them in a test-retest procedure. Psychometric properties were evaluated by determining reliability (internal consistency [Cronbach’s alpha] and test-retest stability [intraclass correlation coefficient]), construct validity (exploratory factor analysis) and content validity.

**RESULTS:**

The development resulted in 60 questionnaire items. Content validity assessment showed that the women overall found it easy to complete the questionnaire. Reliability analyses showed satisfactory results for both direct and indirect measures, with a few exceptions. Factor analyses of the indirect scales showed that items were generally in line with the TPB, but that items related to life as a whole/personal life and items related to work were separated into two different factors.

**CONCLUSION:**

The questionnaire, called the RTW Beliefs Questionnaire, showed promising results and can among women with CMDs be considered useful, especially the scales for direct measures. This questionnaire gives opportunity to identify new potential predictors for RTW.

## 1 Introduction

There are several instruments that measure beliefs about return to work (RTW). However, the questionnaire described in this study is the first based on the Theory of Planned Behaviour (TPB) [1] developed for women on long-term sick leave for common mental disorders (CMDs). This is an important target group, given women’s higher prevalence of long-term sick leave due to CMDs [2]. Furthermore, unfavorable working conditions and having the main responsibility for the family [2] might affect their RTW-beliefs. The questionnaire can complement other questionnaires measuring RTW-beliefs within the area of CMDs, with its potential to identify RTW beliefs underlying behaviour in several layers. The questionnaire can potentially create opportunities to identify previously unknown predictors of RTW among women on long-term sick leave for CMDs. This could be important for RTW-stakeholders as well as for the research community. The developed questionnaire has been used in a previous Swedish study [3] and was found to be useful for identifying determinants of RTW intentions among women. In this study, the development, construction and psychometric evaluation of the questionnaire are described in greater detail to promote a deeper understanding of the questionnaire.

CMDs, which include stress-related disorders, depression and anxiety, are a leading cause of disability worldwide and affect women in particular [2, 4]. Among women in Sweden, long-term sick leave (>60 days) due to CMDs has increased in the proportion of all long-term sick leave from 13% to 41% in the last three decades [5]. Being on long-term sick leave may have negative health consequences, regarding sleep [6], lifestyle habits and psychological well-being [6, 7]. Society, in turn, is economically affected and loses valuable competence, often in the health/social care and school sectors [5, 8]. Hence, it is essential to facilitate RTW for this group – both work resumption and staying at work until stability is achieved [9, 10]. Known facilitators of RTW among individuals on sick leave for CMDs include graded RTW and support from the workplace [11, 12]. This approach is applied in Sweden today [13, 14]. Nevertheless, RTW after long-term sick leave for CMDs remains a major challenge, with the underlying mechanisms still in part unknown. One incompletely investigated aspect is the affected individuals’ perceptions and cognitions about RTW [15], also called beliefs [16]. Beliefs refer to people’s cognitions and are defined as something we perceive to be the truth about, or the probability of, something (e.g., that will happen) [16]. Beliefs about a behaviour, such as RTW, have been shown to be crucial to perform that behaviour [15, 17].

There are questionnaires that include RTW beliefs as defined above, such as readiness to RTW [18], perceived obstacles [19], self-efficacy [19, 20] and RTW expectations [21]. A general pattern is that they have not been developed for women with CMDs, and that they have limitations in relation to RTW. For example, the Readiness to RTW Scale has shown poor predictive validity regarding RTW among individuals on long-term sick leave in general (including among those with CMDs) [22, 23]. Lagerveld et al. developed an RTW self-efficacy scale for individuals with mental health complaints that was able to predict RTW in this group [20] and the score on that scale is known to be a strong predictor of RTW among individuals with CMDs [24, 25]. However, it is unclear if the obstacles to RTW included in that questionnaire are perceived as obstacles by the individuals themselves. Corbière et al. noticed this limitation and developed the RTW Obstacles and Self-Efficacy Scale [19]. This scale first assesses whether or not the individuals perceive a statement to be an obstacle; if they do, they are invited to answer a corresponding question about how capable of overcoming the obstacle they feel they are. Several dimensions of that scale have been shown to be able to predict RTW among individuals with CMDs, but a limitation of the questionnaire is that it concerns RTW to the same workplace [19]. The Fear-Avoidance Beliefs Questionnaire is a well-established instrument used in recent decades among individuals with pain, and has been shown to predict RTW in this group [26]. However, it seems that its predictive properties are related only to the item on expectations [27], which is already a well-known predictor of RTW among individuals with CMDs [21]. Although the importance of RTW expectations is known, there is a knowledge gap regarding how individuals’ RTW expectations can be increased, i.e., what underlies RTW expectations [28]. The questionnaire developed in the present study fills some gaps because it is developed based on the experiences of women with CMDs and the TPB, which is a theory for explaining and predicting human behaviour using a wide range of beliefs at several levels [1, 29]. This theoretical model enables investigation of predictors of RTW, as well as of what underlies RTW intentions (RTW expectations included). Ultimately, the model may provide a framework for interventions to promote RTW.

According to the TPB, human behaviour is not haphazard in nature. Instead, it is determined by various beliefs – hence the term ‘planned behaviour’. The basic idea is that an individual will engage in a behaviour if he/she intends to do so. The intention, in turn, depends on beliefs related to three independent factors: attitude, subjective norms and perceived behavioural control [1]. In other words, if an individual believes (attitude) that performing the behaviour has sufficient advantages, that it conforms to the desires of others (subjective norms, i.e., social pressure) and that he/she possesses enough resources to overcome the barriers to the behaviour (perceived behaviour control), then his/hers intention is strengthened and likelihood of actual performance of the behaviour increases [1]. The factors can be measured both directly and indirectly. Direct measures are worded in a general manner, while indirect measures are developed from the target population and therefore capture specific beliefs about the target behaviour [29, 30].

The theory has been applied to a wide range of behaviours, often health-related behaviours, and has been shown to be robust [30]. However, to date, only a few studies have applied the TPB to RTW. Brouwer et al. [31] showed that attitude, social support and self-efficacy (representing perceived behavioural control) predicted RTW among individuals on sick leave in general. However, the items were not developed to suit their target population. To ensure content validity, the affected group should be involved from the start of development [30]. Dunstan et al. [17] did this with individuals on sick leave for musculoskeletal disorders and showed that attitude, subjective norms and perceived behavioural control were all determinants of RTW intentions and RTW. The instrument they used demonstrated satisfactory psychometric properties. To our knowledge, no questionnaire based on the TPB has been developed for individuals on long-term sick leave for CMDs. We chose to develop the questionnaire for women, as they have higher rates of long-term sick leave due to CMDs in Sweden [4]. Furthermore, it is reasonable to assume that women’s RTW beliefs are influenced by the fact that they often have emotionally demanding jobs (i.e., within the health/social care or school sectors) [5, 8] and primary responsibility for the household [32, 33]. In line with this, Helman [34] suggested that norms and expectations related to gender, created by the culture in society, shape individuals’ beliefs in different ways and Otten et al. [4] suggested the use of a gender-specific approach in mental health research.

In summary, the new questionnaire is a tailored, comprehensive and theory-based measurement of RTW beliefs among women on long-term sick leave for CMDs. Most of its measurement properties are still unknown. Therefore, the present study aimed to describe the development and evaluation of psychometric properties of this questionnaire, designed to measure RTW beliefs among women on long-term sick leave for CMDs.

## 2 Materials and method

**Fig. 1:**
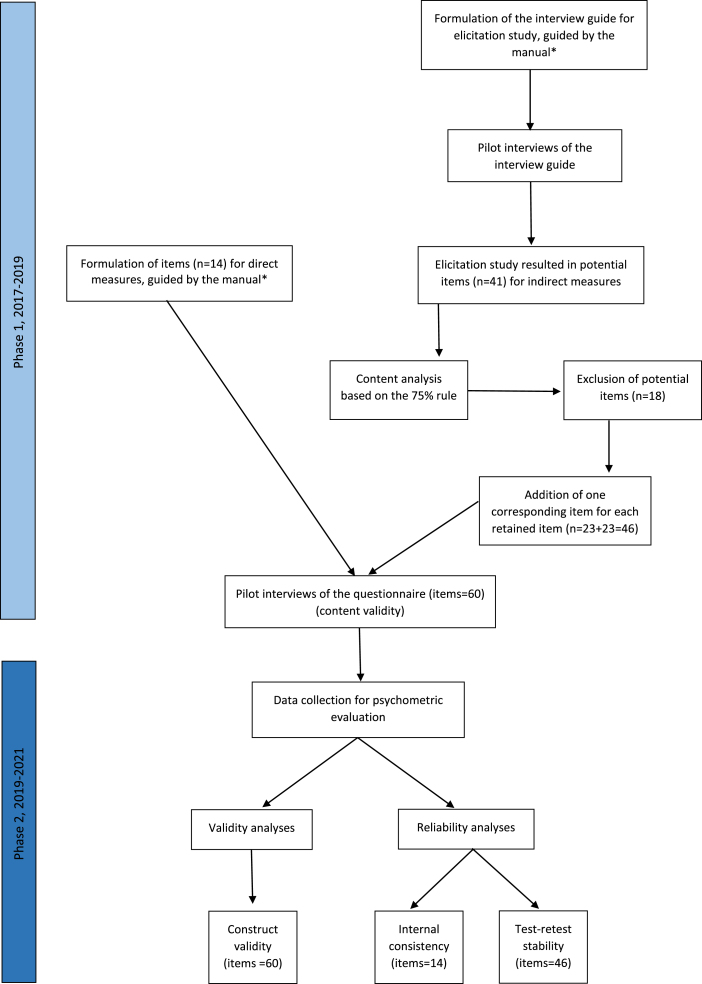
Overview of the process for development (Phase 1) and psychometric evaluation (Phase 2). *Francis et al. [29].

The development and psychometric evaluation of the questionnaire were guided by COSMIN [35] and a manual created by Francis et al. [29], which describes how to construct and psychometrically evaluate questionnaires based on the TPB. Development of the questionnaire was performed in Phase 1 and psychometric evaluation in Phase 2. For an overview of the process, see Fig. 1.

### 2.1 Phase 1: Questionnaire development

#### 2.1.1 Development of items for direct measures

The items used to create scales for direct measures were worded based on recommendations in the manual [29]. The items from the manual were reworded for RTW. The scales were: intention, attitude, subjective norms and perceived behavioural control. Some symptoms of CMDs are common, e.g., tiredness, and we wanted to keep the number of items as low as possible, without losing sight of the purpose of the questionnaire. Therefore, three of the four suggested items for the subjective norms scale were included, as recommended by the manual [29] (see Supplementary material Table A). This resulted in 14 items for direct measures, shown in Table 1.

**Table 1:** Scales (N = 7), subscales (N = 6) and items (N = 60)

Items	Items (n)	Endpoints	Range
**Scales (*n* = 4) for direct measures**	14		
*Intention*	3		
I expect to/want to/intend to RTW^a^ within 3 months			Strongly disagree – Strongly agree	1– 7
*Attitude*	4		
RTW^a^ is for me:		Harmful – Beneficial	1– 7
		Worthless – Useful	
		Good – Bad	
		Pleasant – Unpleasant	
*Subjective norms*	3		
It is expected of me/I feel under social pressure to/people who are important to me want me to RTW^a^		Strongly disagree – Strongly agree	1– 7
*Perceived behavioural control*	4		
I am confident that I could/the decision is beyond my control/whether or not I RTW^a^ is entirely up to me		Strongly disagree – Strongly agree	1– 7
RTW^a^ is for me:		Easy – Difficult	
**Scales (*n*** = **3) for indirect measures**	**46**		
*Subscales (n* = *2) for behavioural beliefs*	18		
Advantages: If I RTW^a^ I will: feel more like I’m part of a social context/get improved daily routines/get improved health/feel that life is more meaningful/get an improved economy/feel secure (if at the same working place)/maintain or increase my feeling of competence	7	Strongly disagree – Strongly agree	1– 7
Disadvantages: Increased symptoms/won’t manage the same achievements as before	2		
Evaluation of outcome: To (advantage/disadvantage) is for me:	9^b^	Not important at all – Very important	– 3– +3
*Subscales (n* = *2) for normative beliefs*	14		
Supporters: Family and relatives/friends/Social Insurance Agency/colleagues/employer	5	Strongly disagree – Strongly agree	– 3– +3
Non-supporters: Family and relatives/friends (if health gets worse)	2		
Motivation to comply: That (supporters/non-supporters) want me to RTW^a^ is:	7^c^	Not important at all – Very important	1– 7
*Subscales for control beliefs (n* = *2)*	14		
Facilitators: Getting support from friends, family and relatives/healthcare staff/well-adapted work tasks I can perform at my own pace is:	3	Unlikely – Likely	1–7
Barriers: Employer’s, colleagues’ and/or stakeholders’ unreasonable demands/my own unreasonable demands/lack of support from the surroundings/deteriorating health is/are:	4		
Influence on outcomes: (Facilitator/barrier) makes me/it	7^d^	Less motivated – More motivated (facilitators)	– 3– +3
More difficult – Easier (barriers)

^a^RTW: return to work. Women on full-time sick leave responded to ‘return to work’ and women who were working to some extent responded to ‘stay at work’. ^b^Multiplied by behavioural beliefs. ^c^Multiplied by normative beliefs. ^d^Multiplied by control beliefs.

#### 2.1.2 Development of items for indirect measures – elicitation study

The interview guide (Table 2) was developed through consulting the manual [29] and was tested on five individuals, before the elicitation study (an interview study to generate items), without any changes being made. Women met the criteria for the elicitation study if they were≥18 years, on full- or part-time sick leave for CMDs (ICD-10-SE codes: F30– F48 [36]) for≥2 months. They also had to be able to read, write and speak Swedish. Criteria for exclusion were severe mental illness, unemployment or on sick leave > 2 years. In December 2017, the Swedish Social Insurance Agency (SIA) invited 150 women on long-term sick leave for CMDs, who lived in a county in central Sweden, to participate in the elicitation study. A total of 25 women expressed interest in doing so. However, five changed their minds for various reasons, e.g., they did not feel they had the energy to participate in an interview after all. Hence, a total of 20 women were included in the elicitation study.

**Table 2:** Interview guide for the elicitation study

**The interview questions**
**Behavioural beliefs**
What do you believe are the advantages of returning to work^1^?
What do you believe are the disadvantages of returning to work?
Is there anything else you associate with returning to work?
**Normative beliefs**
Are there any individuals or groups who would approve of you returning to work?
Are there any individuals or groups who would disapprove of you returning to work?
Based on individuals or groups, is there anything else you associate with returning to work?
**Control beliefs**
What factors or circumstances enable you to return to work?
What factors or circumstances make it difficult or impossible for you to return to work?
Are there any other issues that come to mind when you think about returning to work?
**Additional question**
Is there anything else you associate with returning to work?

^1^If the women worked to some extent, ‘return(ing) to work’ was replaced with ‘stay(ing) at work’, throughout the interview.

Interviews were conducted by the first author in January– May 2018. The women were asked about their behavioural, normative and control beliefs regarding RTW, as well as about background information, such as age, diagnosis and extent of sick leave. They listed their beliefs in writing and talked about them. A manifest qualitative content analysis [37] was conducted by the authors to determine beliefs that should become items for indirect measures. Behavioural, normative and control beliefs were analysed separately. The beliefs stated in the interviews were grouped based on their similarities and differences and named based on their content. The 75% rule recommended by Francis et al. [29] was used, i.e., choosing the most frequently mentioned groups that together constituted around 75% of the stated behavioural, normative and control beliefs, respectively. For example, there were totally 142 stated behavioural beliefs and 24 (16.9%) of those were about belonging to a social context and 13 (9.2%) were about routines. The shares of the most frequently mentioned groups were added together until the sum was about 75%. The names of these groups were retained, to become items for indirect measures. The analysis resulted in 23 items divided across the three scales for indirect measures: behavioural beliefs (*n* = 9), normative beliefs (*n* = 7) and control beliefs (*n* = 7). Within each scale, there were two subscales in line with the TPB: advantages and disadvantages (behavioural beliefs), supporters and non-supporters (normative beliefs) and facilitators and barriers (control beliefs). The items were worded with guidance from the manual [29]. When the manual offered multiple options for wording or number of items, the authors discussed and agreed on what was best suited for the target population and target behaviour (RTW). For each item, a corresponding item was created to assess the outcome evaluation (behavioural beliefs), the motivation to comply (normative beliefs) and the impact on the behaviour (control beliefs). For example: ‘If I return to work/stay at work, life will feel more meaningful: strongly disagree – strongly agree’ (behavioural belief, item range 1– 7) and ‘Perceiving meaningfulness in life is, for me: not important – very important’ (evaluation of outcome, item range – 3 to +3). Hence, a total of 46 items for indirect measures were included, see Table 1.

#### 2.1.3 Pilot interviews of the entire questionnaire to evaluate content validity

The last step was to let women test the questionnaire. In the spring of 2019, interviews were carried out with five women from the elicitation study, as recommended by Francis et al. [29]. The women were purposively chosen to achieve differences in age and diagnoses. Four women participated in face-to-face interviews and one responded by e-mail. The interviews were based on the following questions recommended by the manual [29]: Are any items ambiguous or difficult to answer? Does the questionnaire feel too repetitive? Does it feel too long? Does it feel too superficial? Are there any annoying features in the wording or formatting (including endpoints)? Furthermore, inconsistent responses to the questionnaire were looked for, as they might indicate that changes in the response endpoints were problematic (this could not be done for the woman who answered via e-mail). The authors then discussed the findings.

#### 2.1.4 Description of the final questionnaire

Table 1 provides an overview of the scales and items. The questionnaire consisted of 60 items divided into seven scales and six subscales. The items were worded to be suitable for women on either full-time or part-time sick leave, e.g. “People who are important to me want me to return to work/stay at work”. Women who were on full-time sick leave focused on RTW and women that worked to some extent focused on staying at work when responding. Responses were rated on 7-point scales, unipolar (1– 7) or bipolar (– 3– +3). The items from the different scales were all mixed together except those for direct measures of attitude, which were grouped for theoretical reasons, i.e. the items belonged to the same main item. Background questions were included as well. The questionnaire in its original form is published in a previous study [3].

### 2.2 Phase 2: Psychometric evaluation

#### 2.2.1 Participants, setting and procedure

The women met the criteria for the psychometric evaluation if they were≥18 years and on full- or part-time sick leave for CMDs for at least the preceding two months (ICD-10-SE codes: F32– F33, F35– F48 [36]). They also had to be able to understand written Swedish. Criteria for exclusion were severe mental illness, unemployment or sick leave > 2 years. The sample size needed was determined to be around 300 based on recommendations from Polit and Beck [38] regarding the required sample size for an estimated effect size of 0.35, a power of 0.8, and *α*= 0.05. These estimations are common within nursing research [38]. We did also take into account the number of participants needed for a factor analysis, where 300 is considered as good [39]. Based on previous research among a similar sample [40], we did expect a response rate of around 30%. The researchers prepared invitations, each with a unique code. In October 2019 and January 2020, SIA identified a total of 1,196 potential participants in two counties in central Sweden and sent them the invitations. The women could choose to complete the questionnaire on paper or electronically. Two reminders were sent out from SIA at 2-week intervals. A total of 371 questionnaires were returned to the researchers (367 on paper and four electronically), of which 89 were excluded. Reasons for exclusion were, no longer being on sick leave (e.g. have RTW full time or retired), unemployment, or an incomplete questionnaire. Hence, 282 women were included (response rate 23.5%). Subsequently, a test-retest procedure was carried out with an interval of around three weeks, in conjunction with a 1-year follow-up. Test-retest does generally not require more than 50 participants [35], why we considered it reasonable to invite the first 50 women who responded to the 1-year follow-up to the test-retest. The retest followed the same procedure as the former test. In total, 35 women participated. It is desirable that the conditions, on our case RTW-beliefs, has not changed between the test and the retest [35]. Although there are no guarantees, the conditions were considered to be approximately the same on the two test occasions. For example, it is reasonable to assume that the women still had the same supporting people around them after three weeks.

#### 2.2.2 Preparation of the scales for data analyses

**Fig. 2:**
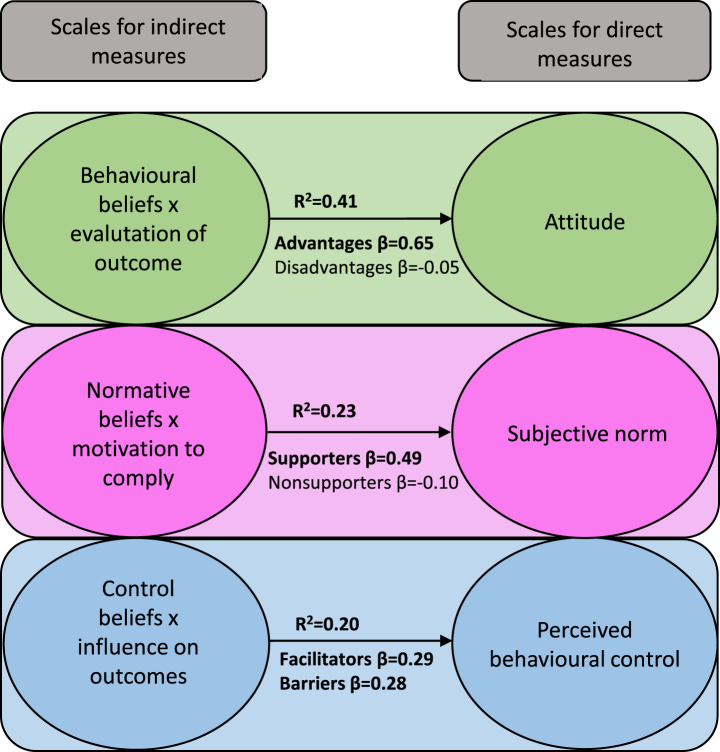
Associations between indirect and direct measures (*n* = 268– 271). Model fit regarding indirect and direct measures for attitude: *F*(2,268) = 93.86, *p* < 0.001, for subjective norms: *F*(2,265) = 41.88, *p* < 0.001, and for perceived behavioural control: *F*(2,266) = 33.52, *p* < 0.001. Bold numbers represent significant values (*p* < 0.001).

In preparing the scales for direct measures, the items with negatively worded endpoints on the right-hand side were recoded, so that high scores consistently reflected the same direction (i.e., stronger intention, more positive attitude, stronger social pressure, stronger perceived behavioural control). Then, the mean of each scale was calculated, producing overall scores. When calculating the indirect measures, each item was multiplied by its paired item, as illustrated in Fig. 2. Thus, among the 46 items for indirect measures, 23 products were created with the range – 21 to +21 (1– 7×– 3– +3), representing the perceived importance of the belief about RTW (a higher value indicated a higher perceived importance). The products were added together to create an overall score for each scale and subscale for indirect measures. Descriptive statistics for the scales such as mean and standard deviation is presented in the previous study [3].

#### 2.2.3 Data analysis

Descriptive statistics: Frequency and percent were calculated for the demographic data for the total sample and the retest sample, respectively.

Reliability: The scales for direct measures are reflective, which means that they represent the same underlying construct, i.e. that the items are assumed to be correlated with each other [35]. Internal consistency (Cronbach’s alpha) was therefore calculated for each of the scales for direct measures. This is partly presented in a previous study [3]. Desirable score for alpha is according to Francis manual [29] 0.6 or above. Internal consistency was not relevant to calculate for the scales of indirect measures because of their formative nature [35], i.e. that the items are not expected to correlate with each other. Instead, test-retest stability was calculated for these scales using intraclass correlation coefficients (ICCs) with a 2-way mixed model and absolute agreement type [41]. According to Terry and Mae [41], ICC values less than 0.50, between 0.50 and 0.75, 0.75 and 0.90, and greater than 0.90 means poor, moderate, good and excellent reliability, respectively.

Validity: To assess content validity, data from the pilot interviews (*n* = 5) were structured into a table based on the questions proposed by Francis et al. [29] and then discussed between the authors to agree on any changes to the questionnaire. Standard multiple regression analysis was used between the indirect and direct scales. In each of the three regression models, the direct scale was entered as the dependent variable and the two subscales within each indirect scale were entered as independent variables. Assumptions for regression analysis were met, i.e., variance inflation factor values were≤1.06, indicating no presence of multicollinearity [42]. The residual plots showed a linear pattern. There were a few outliers in the subjective norms scale. However, eliminating them did not yield any change in the result.

To study the construct validity of the scales for indirect measures, exploratory factor analyses (EFA) were conducted using principal axis factoring and oblimin rotation with Kaiser normalisation. EFA are useful in an initial stage of creating a questionnaire, to get a first overview of its construction [43]. Principal axis factoring is preferable when the questionnaire is primarily intended to be used on similar populations as in the testing [43]. The oblimin rotation method allows factors to be correlated, which often produces more accurate results in research of human behaviour [39]. The number of factors was determined with eigenvalue > 1. Assumptions for EFA [39, 42] were met, i.e., all Kaiser-Meyer-Olkin Measures of Sampling Adequacy were > 0.6, and Bartlett’s tests showed significance (*p* < 0.001). Moreover, several correlation coefficients (Pearson’s r) between items were > 0.3, indicating that items were correlated enough for a factor analysis to be meaningful [39]. In accordance with the literature [42], linearity was tested by creating a series of scatterplots between some of the items; these did not show any pronounced deviation from linearity.

Missing values within the scales did not exceed 2.5%, and overall, 1% of the values were missing. A few of the missing values were due to that items about the employer and/or colleagues were unsuitable for self-employed women, and items about friends were unsuitable for women that felt they did not have any friends. To deal with missing data, ‘exclude cases pairwise’ was applied in the regression analyses and the EFA. The significance level was set to *p* < 0.05. All analyses were conducted using IBM SPSS Statistics version 27.0 [44].

## 3 Results

### 3.1 Phase 1: Elicitation study and pilot interviews

#### 3.1.1 Elicitation study

The 20 women included in the elicitation study were aged 28– 63 years (median 45 years). At the time of the interviews, three of the women were working full time, while three were participating in work-oriented rehabilitation or combining work with parental leave/leave of absence. Fourteen were still on long-term sick leave. The most common diagnosis was a stress-related disorder (*n* = 13), and the most common professions were in the health/social care or school sectors (*n* = 12). Having a poor working environment was a frequently reported cause of sick leave (*n* = 14). Other reasons were traumatic events in private life or high demands on oneself. Most women (*n* = 14) had previous experience ofCMDs.

Forty-one potential items for indirect measures were derived from the interviews, equally divided between the three scales for indirect measures. Regarding behavioural beliefs, i.e., the advantages and disadvantages of RTW, the women mentioned almost as many advantages as disadvantages. However, when applying the 75% rule, most advantages were retained and most disadvantages were excluded. The retained advantages concerned different areas in life, while the retained disadvantages only concerned symptom burden. For normative beliefs, i.e., supporters and non-supporters of RTW, the women mentioned more supporters of RTW than non-supporters, and when the 75% rule was applied, more supporters than non-supporters were retained. A general pattern for subjective norms was that the closest associates (e.g., family/friends) were retained and more distant associates (i.e., superficial acquaintances or society as a whole) were excluded. Regarding control beliefs, i.e., barriers to and facilitators of RTW, the women mentioned more facilitators of RTW than barriers to it, but after the analysis according to the 75% rule [29], more barriers than facilitators were retained. Overall, perceived behavioural control concerned (reasonable) demands. Retained items for indirect measures are presented in Table 1, and the excluded beliefs for potential items are provided in the supplementary material in Table B.

#### 3.1.2 Pilot interviews to determine content validity

On the whole, women were of the opinion that the items were clear and easy to answer, and most of their opinions were about ambiguities in the background questions. However, one woman felt that she had to concentrate because the endpoints sometimes changed. Another woman felt that some items were difficult to respond to if one thought about them from multiple perspectives, but easy to respond to when scored based on what first came to mind. Three of the women felt that the questionnaire was repetitive, but this was described as positive, because this meant it required less energy to complete. Another opinion was that ‘meaningfulness’ and ‘routines’ felt like clichés. The structure was perceived as confusing by two women because the items were presented in groups of three even though they had nothing in common. When reviewing the questionnaires afterwards, it seemed that one woman might not have noticed the change in endpoint direction in the middle of the questionnaire. None of the women felt that the questionnaire was too long; it took about 15 minutes to complete. In summary, the pilot interviews resulted in some changes in structure and additional clarification of the background questions.

### 3.2 Phase 2: Psychometric evaluation

The women who participated in the psychometric evaluation (*n* = 282) were aged 22– 66 years (mean 45 years, SD 11.2). A majority (*n* = 176) were on part-time sick leave and the rest were on full-time sick leave. Half of the women worked in the health/social care or school sectors (*n* = 140). Stress-related disorders were the single most commonly reported diagnosis. The women who participated in the test-retest (*n* = 35) had similar characteristics. For full participant characteristics, see Table 3.

**Table 3:** Self-reported characteristics of the total sample (*n* = 282) and the retest sample (*n* = 35)

	Total sample	Retest sample
Variables	Frequency (%)	Frequency (%)
*Country of birth*		
Sweden	263 (93.3)	34 (97.1)
Other^1^	18 (6.4)	1 (2.9)
Not available	1 (0.3)	0
*Number of children living at home*		
≥1 children living at home	136 (48.2)	15 (42.9)
No children living at home^2^	142 (50.4)	20 (57.1)
Missing	4 (1.4)	0
*Marital status*		
Living with partner/parents^3^	207 (73.4)	15 (71.4)
Living alone with/without children	75 (26.6)	10 (28.6)
*Education*		
Elementary school	27 (9.6)	0 (0.0)
Upper secondary school	126 (44.7)	14 (40.1)
Post-upper secondary school	4 (1.4)	1 (2.9)
University	125 (44.3)	20 (57.1)
*Diagnosis*		
Stress-related disorder	126 (44.6)	11 (31.3)
Depression	62 (22.0)	4 (11.4)
Anxiety	7 (2.5)	1 (2.9)
A combination of a stress-related disorder and/or anxiety and/or depression	71 (25.2)	17 (48.6)
Other^4^	3 (1.1)	1 (2.9)
Missing	13 (4.6)	1 (2.9)
*Professional sector*		
Health care, schools and social service	140 (49.7)	18 (51.4)
Administrative work	40 (14.2)	4 (11.4)
Sales and services	33 (11.7)	4 (11.4)
Leading position or self-employed	27 (9.6)	3 (8.6)
Industry workers and engineers	11 (3.9)	3 (8.6)
Other^5^	31 (10.9)	3 (8.6)
*Other health problems (comorbidity)*		
Yes^6^	95 (33.7)	19 (54.3)
No health problems except CMDs	185 (65.6)	16 (45.7)
Missing	2 (0.7)	0 (0.0)
*Extent of sick leave (%)*		
100	106 (37.6)	12 (34.3)
25– 75	176 (62.4)	23 (65.7)

^1^Half from Europe and half from other parts of the world. ^2^More than half had no children. ^3^Six lived alone, but had a partner. Two still lived with their parents. ^4^Obsessive compulsive disorder or neurodevelopmental disorders. ^5^Such as creative or media-related jobs. ^6^Most often musculoskeletal disorders.

#### 3.2.1 Reliability

Results of the reliability tests are shown in Table 4. Internal consistency for the scales for direct measures varied in the range 0.43– 0.92. The intention and attitude scales had the highest internal consistency. The subjective norms and perceived behavioural control scales showed low internal consistency. Inter-item correlations did not support removal of any items in the subjective norms scale, but showed that the item ‘Whether I return to work/stay at work is entirely up to me’ in the perceived behavioural control scale was weakly correlated with the other items in the scale as well as with the total scale score (Spearman’s rho = 0.08). Elimination of that item increased the alpha value from 0.48 to 0.60. Test-retest stability for the scales for indirect measures showed moderate to excellent stability (ICC 0.70– 0.92), with attitude showing the strongest stability and subjective norms the weakest.

**Table 4:** Reliability of the subscales

	Internal consistency^a^	Test-retest stability^b^
	(*n* = 282)	(N = 35)
*Direct measures*		
Intention	0.92^c^	
Attitude	0.85^c^	
Subjective norms	0.43^c^	
Perceived behavioural control	0.48 (0.60^c,d^)	
*Indirect measures*		
Behavioural beliefs×evaluation of outcome		0.92^e^
Normative beliefs×motivation to comply		0.70^e^
Control beliefs×influence on outcomes		0.86^e^

^a^Cronbach’s alpha. ^b^Intraclass correlation coefficient. ^c^Reported in a previous study (2). ^d^After removing the item that showed the lowest inter-item correlation. ^e^Significant at the 0.001 level.

#### 3.2.2 Validity

Standard multiple regression analyses showed that the direct and most of the indirect scales were significantly associated with each other, which confirm the construct validity of the model. The analyses demonstrated that advantages (indirect subscale for attitude) and supporters (indirect subscale for subjective norms) were significantly associated with attitude and subjective norms, respectively, but that disadvantages and non-supporters were not. However, barriers and facilitators were both significantly associated with the direct scale of perceived behavioural control. Associations between direct and indirect scales are shown in Fig. 2.

The EFA on the scales for indirect measures showed that factors were generally in line with the theory, with one deviating pattern: Advantages (subscale within indirect measures for attitude) and supporters (subscale within indirect measures for subjective norms) were divided into two factors each, one related to life as a whole/personal life and one related to work. Therefore, three-factor solutions were shown for these scales. Also, the item about improved economy (advantage within indirect measures for attitude) loaded strongest together with disadvantages. The results of the EFA are presented in Table 5.

**Table 5:** Exploratory factor analyses on the subscales for indirect measures (*n* = 274– 281)

**Behavioural beliefs×evaluation of outcome** (indirect scale for attitude)	Identified factors and item loadings^a^		
*Advantages related to life as a whole*	*Disadvantages*	*Advantages related to work life*
*Advantages of RTW^*b*^*			
Improved health	**0.598**	–0.154	–0.255
Improved daily routines	**0.837**		
Meaningfulness	**0.691**	0.207	–0.151
Social context	**0.746**		
Security (if the same workplace)		–0.107	**–0.744**
Feeling of competence	0.126	0.276	**– 0.663**
Improved economy	0.246	**0.387** ^c^
*Disadvantages of RTW*			
Increased symptoms	–0.274	**0.459**	
Won’t manage the same achievements as before		**0.373**	–0.200
Explained variance (%)	**35.37**	**7.76**	**5.72**
Total variance explained (%)	**48.80%**		
**Normative beliefs×motivation to comply** (indirect scale for subjective norms)	*Supporters in personal life*	*Non-supporters*	*Supporters in work life*
*Supporters of RTW*		
Family/relatives	**0.905**		
Friends	**0.906**		
Employer			**– 0.870**
Colleagues		0.109	**– 0.779**
Social Insurance Agency	**0.361**		–0.233
*Non-supporters of RTW*		
Family and relatives (if health gets worse)		**0.760**	
Friends (if health gets worse)		**0.617**	
Explained variance (%)	**35.08**	**14.42**	**10.65**
Total variance explained (%)	**60.20%**		
**Control beliefs×influence on outcomes** (indirect scale for perceived behavioural control)	*Barriers to RTW*	*Facilitators of RTW*
*Facilitators of RTW*		
Support from friends, family and relative		**–0.783**	
Support from healthcare staff		**– 0.742**	
Well-adapted work tasks I can perform at my own pace		**– 0.496**	
*Barriers of RTW*			
Employer’s, colleagues’ and/or stakeholders’ unreasonable demands	**0.698**		
Lack of support from the surroundings	**0.610**		
Deteriorating health	**0.581**	–0.135	
My own unreasonable demands	**0.455**		
Explained variance (%)	**27.07**	**13.69**	
Total variance explained (%)	**40.76%**		

^a^Oblimin rotation with Kaiser normalisation. Loadings below 0.1 are not shown in the table. Bold numbers represent the strongest factor loading for the item. ^b^Return to work. ^c^Should be considered to belong to the first factor.

## 4 Discussion

The questionnaire, called the ‘RTW Beliefs Questionnaire’, included 60 items: 14 for direct measures and 46 for indirect measures. Content validity assessment based on the pilot interviews showed that women overall were of the opinion that the questionnaire was easy to understand and complete. The psychometric evaluation showed promising results overall, though there are some aspects to consider. Reliability analyses (internal consistency and test-test stability) were found to be satisfactory for both direct and indirect measures, except in regard to the direct scale for subjective norms. Scales for direct measures and subscales for indirect measures were in most cases significantly associated with each other, which confirms the construct validity of the TPB model. Construct validity of the indirect scales, studied using EFA, showed that items were generally in line with the TPB. However, in the attitude and subjective norms scales, items related to life as a whole/personal life and items related to work were separated into two different factors.

The target population was involved in the generation of items from the very beginning, revealing the breadth of beliefs underlying intentions and behaviours [30]. Evaluation of content validity according to Francis et al. [29] did not show any major problems with the questionnaire. Despite this, there were some concerns that were not captured in the pilot interviews, for instance because the items on employer, colleagues and friends were impossible for a few women to answer. In future research, it is important to be aware of the weaknesses of items including ‘others’ (e.g., employer, colleagues) and perhaps choosing a sample for which these items are appropriate, e.g. not self-employed women. Furthermore, applying the 75% rule might constitute a threat to content validity as several potential items were excluded from the analysis. It is known that questionnaires based on the TPB may have problems with content validity, as a result of wanting to keep the number of items down, especially if the target behaviour (in this case RTW) is complex [30]. Because the 75% rule is not required in development of TPB-based questionnaires [46], further research could include excluded potential items (see Table B in Supplementary materials) to investigate whether any of them enhance the psychometric properties of the scales for indirect measures. For example, there are theoretical reasons to assume that coping skills and meaningfulness are important when facing challenges in life [47, 48].

The direct scales for intention and attitude showed good internal consistency. However, the scale for subjective norms showed low internal consistency. This may be explained by the few items or that the scale was heterogeneous [49]. The latter is interesting to consider, as the EFA on the scales for indirect measures showed a distinction between social pressure from actors in private life and work life [49]. Perhaps this distinction is a reason for the scale’s heterogeneity. Another explanation is that one of the recommended items was left out (see Table A in supplementary materials) in the development phase due to its similarities to another item on the same scale. In future research, re-inclusion of this item should be considered. The direct scale for perceived behavioural control also showed a low alpha value. This is explained by the fact that the item ‘Whether I return to work/stay at work is entirely up to me’ showed almost no correlation with the other items, and elimination of that item increased alpha value to an acceptable level (0.60) for this kind of questionnaire [29]. This item differs from the others in the scale because it asks about total control over RTW. There are several possible reasons why this item does not belong with the others. First, it may be due to the nature of CMDs, i.e., that symptoms are perceived as barriers to RTW, as shown in previous research [7], and cannot fully be controlled. Another possible explanation is that the women felt that other stakeholders could affect RTW in a direction they did not desire. For example, SIA could reject an application for prolonged sick leave, resulting in a woman having to RTW even if it was not her own decision. We suggest elimination of this item in future research among similar samples.

The regression analyses demonstrated that scales for indirect and direct measures were significantly associated with each other, which confirms the construct validity of the model. The exceptions from this were the scales for disadvantages (indirect subscale for attitude) and non-supporters (indirect subscale for subjective norms), which were not significantly associated with the direct scales of attitude and subjective norms. Previous studies that have used the TPB among similar samples [17, 31] have not investigated the relationship between indirect subscales and the direct scales. This new finding may mean that beliefs that argue for RTW are more important for attitudes and social pressure to RTW, than beliefs that speak against RTW. This might be considered in a future process of shortening the questionnaire, which is desirable because it might be too comprehensive based on the response rate and the nature of CMDs (e.g. difficulties to concentrate and exhaustion).

Regarding the scales for indirect measures, the EFA suggested a third factor regarding advantages and supporters, one related solely to work life. This indicates that there is a perceived distinction between personal life and work life for women on long-term sick leave due to CMDs. Interestingly, this pattern was not seen among individuals with musculoskeletal disorders [17]. However, it is known from previous literature that RTW after sick leave due to CMDs is based on an interplay between personal life and work life [50], and that interventions aiming to facilitate RTW are more successful if they consider both areas [11, 12]. Hence, the division suggested by the EFA seems logical, and we suggest that it should be considered when using indirect measures as factors in future research for prediction of attitude and subjective norms. Furthermore, the explained variance differed greatly between the scales for indirect measures. Attitude and perceived behavioural control had a variance below the recommended limit of 50% in human sciences [39, 43]. This makes them unsuitable for use as factors in their current form; further psychometric testing among women is needed to evaluate the issues. Further studies with samples of both men and women could also contribute to the development of these scales. In contrast, the three factors within indirect measures for subjective norms explained 60.2% of the variance, which is good [39, 43].

This development and initial testing of the questionnaire show interesting results that, in the long run, might contribute to research aiming to explain and predict RTW among women after long-term sick leave due to CMDs. It is important to notice though that this questionnaire does not state how the outcome RTW should be measured. In this study, we have investigated several aspects of reliability and validity. Nevertheless, it is still in a premature phase and would benefit from further psychometric testing recommended by COSMIN checklist [35], such as construct validity using confirmatory factor analysis, concurrent validity, deeper investigation of content validity (e.g. if items are an adequate reflection of the construct to be measured), and not least predictive validity. The scales for indirect measures should be further tested on women because they are developed from those, while the direct measures should be able to be used more generally.

### 4.1 Limitations of the study

The present study has some limitations. First, the low response rate threatens the generalisability of the results. Unfortunately, a non-response analysis could not be performed, as we did not have access to the SIA register data for the women who did not respond to the study invitation. However, the characteristics of the participants showed that the distribution of their educational level, diagnoses and professions was similar to that among all women on sick leave due to CMDs in Sweden [4]. International comparisons are difficult to do because of different diagnostic systems and registers in different countries, but there are indicators of similarities regarding diagnosis [51] and professions [52] as regards individuals on sick leave in other countries. Furthermore, SIA identified women based on their main cause of sick leave. This does not rule out the presence of comorbidity, i.e., it is possible that chronic or severe mental illness occurred in the sample as well as neurodevelopmental disorders. Moreover, three women from the elicitation study worked full time at the time of the interviews, but all women in the psychometric evaluation were on sick leave. The generation of items might have been somewhat differently if all women in the elicitation study had still been on sick leave. The present study was based on a heterogeneous sample in some respects, e.g., some women were on full-time sick leave, while others worked almost full time. It is reasonable to assume that these women related to the items in different ways, which may have resulted in weak psychometric properties. Furthermore, even if all women had a work to return to, it is unknown if there were other factors beyond their control that affected their beliefs, such as changed circumstances at work. Regarding Phase 2, the representativeness can be questioned, as the sample consisted of women in a relatively small and rural area in Sweden with high and rapidly increasing rates of CMDs. It is furthermore reasonable to assume that women who were most affected by CMDs did not participate, because common symptoms are concentration difficulties and fatigue, which may make it difficult to complete a questionnaire. Last, the choice of only including women can be considered both as a limitation and a strength. On one hand, it makes the questionnaire more limited regarding its use. On the other hand, it is specifically adapted to the group who have the longest sick leaves due to CMDs, which means that it has greater potential to increase the understanding of women’s RTW-process among RTW-stakeholders and researchers.

## 5 Conclusions

This development and initial psychometric evaluation of the RTW Beliefs Questionnaire based on the TPB showed promising results overall. In particular, scales for direct measures may have the potential to be valuable in future research as predictors of RTW, after amendments regarding subjective norms and perceived behavioural control to increase their internal consistency. The indirect measures revealed new aspects of potential importance for RTW among women on long-term sick leave. For example, the exploratory factor analysis showed a distinction between personal life and work life regarding attitude towards RTW and social pressure to RTW. However, indirect scales for attitude and perceived behavioural control should be analysed further among women to enhance construct validity. Nevertheless, this new theory-based questionnaire enables investigation of beliefs of importance to RTW for women on long-term sick leave due to CMDs that have not been investigated previously.

## Ethical approval

The study followed the principles of the WMA Declaration of Helsinki and was approved by the Swedish Ethical Review Authority (Reg. no. 2017/366 and Reg. no. 2020-02587).

## Informed consent

All participants gave written informed consent at the time of the data collection. They received written information about the purpose and content of the study, assurance of confidentiality and were told that participation was voluntary and that they could withdraw their consent at any time without justification. The participants were also informed that the study was a collaboration between the university and Swedish Social Insurance Agency and were assured that their right to sick leave benefits would not be affected by their participating or not participating in the study.

## Conflict of interest

None to report.

## Supplementary Material

Supplementary Material
