# Supplementary material for: Development and psychometric evaluation of a theory-based questionnaire measuring women’s return-to-work beliefs after long-term sick leave for common mental disorders
Source: Work. 2023 Sep 11;76(1):109–24. doi: 10.3233/WOR-220301 (PMC10578269; doi:10.3233/WOR-220301)
Supplement: Supplementary Material [file wor-76-wor220301-s001.docx]

**Table A.** Chosen and left out item within direct scale for subjective norm

| **Similar items** |  |
| --- | --- |
| “People who are important to me want me to…” | Chosen |
| “Most people who are important to me think that I should…” | Left out |

**Table B.** Excluded beliefs for potential items for indirect measures

| **Behavioural beliefs** | **Normative beliefs** | **Control beliefs** |
| --- | --- | --- |
| *Advantages*  Feel needed | *Supporters*  Acquaintances, like neighbours  Care recipients/clients  Healthcare staff  Society at large | *Facilitators*  Decreased demands  Support from colleagues  Support from authorities  Coping  Meaningfulness |
| *Disadvantages*  Negative treatment from others  Difficulty changing patterns  Poor leadership  Difficulty combining working life and private life | *Non-supporters*  Colleagues  Employer  Healthcare staff | *Barriers*  Poor work environment |
